# A Mixed Phytogenic Modulates the Rumen Bacteria Composition and Milk Fatty Acid Profile of Water Buffaloes

**DOI:** 10.3389/fvets.2020.00569

**Published:** 2020-08-26

**Authors:** Faiz-ul Hassan, Hossam M. Ebeid, Zhenhua Tang, Mengwei Li, Lijuan Peng, Kaiping Peng, Xin Liang, Chengjian Yang

**Affiliations:** ^1^Key Laboratory of Buffalo Genetics, Breeding and Reproduction Technology, Ministry of Agriculture and Guangxi Buffalo Research Institute, Chinese Academy of Agricultural Sciences, Nanning, China; ^2^Institute of Animal and Dairy Sciences, University of Agriculture, Faisalabad, Pakistan; ^3^Dairy Science Department, National Research Centre, Giza, Egypt

**Keywords:** mixed phytogenic, rumen bacteria, rumen fermentation, milk yield, fatty acids, buffalo

## Abstract

This study was aimed to evaluate the effect of a mixed phytogenic (MP) on rumen bacteria and their potential association with rumen fermentation and milk yield parameters in water buffaloes. Twenty Murrah buffaloes were fed a basal diet (consisting of maize silage, brewers' grains, and concentrate mixture) for 6 weeks supplemented with 0 (control), 15 (MP15), 25 (MP25), and 35 (MP35) g of mixed phytogenic/buffalo per d. The mixed phytogenic contained fennel (seeds), ajwain (seeds), ginger (tubers), *Swertia chirata* (leaves), *Citrullus colocynthis* (fruit), turmeric, fenugreek (seeds), *Terminalia chebula* (fruit), licorice (roots), and *Phyllanthus emblica* (fruit) in equal quantities. After 2 weeks of adaptation, daily milk yield, and weekly milk composition were recorded. On the last day of the experiment (d 42), rumen contents were collected to determine rumen fermentation parameters and bacterial diversity through 16S rRNA sequencing. Results revealed no change in dry matter intake, milk yield and rumen fermentation parameters except pH, which increased (*P* = 0.029) in response to MP supplementation. The mixed phytogenic increased (*P* < 0.01) milk fatty acids (C4 to C14:0) in MP15 only. The milk C16:1 content and its unsaturation index were higher (*P* < 0.05) in MP35 as compared to the control and other treatments. Furthermore, C18:3n3 was higher (*P* < 0.05) in the control, MP15, and MP25, as compared to MP35. Supplementation of MP tended to increase (*P* = 0.095) the Shannon index of bacterial alpha diversity and a difference (*P* < 0.05) among treatment groups was observed in beta diversity. Feeding MP increased the *Firmicutes, Proteobacteria*, and *Spirochaetes* but decreased *Bacteroidetes* numerically. In addition, the dominant genus *Prevotella* decreased in all treatment groups while *Pseudobutyrivibrio, Butyrivibrio*, and *Succinivibrioanceae* increased numerically in MP25 and MP35. The mixed phytogenic promoted groups of rumen bacteria positively associated with milk and fat yield. Overall, our study revealed 14 positive correlations of rumen bacteria with milk yield and eight with rumen fermentation parameters. Our findings reveal substantial changes in the rumen bacteriome composition and milk fatty acid content in response to MP but these results should be interpreted carefully, as the sample size of our study was relatively small.

## Introduction

Gut microbes perform major digestive and metabolic activities to derive energy from nutrient components of the diet and are considered one of the crucial factors affecting the feed conversion efficiency of ruminants. During rumen fermentation, fermentable dietary components are broken down into volatile fatty acids (VFA) and microbial protein (MCP) is synthesized. Volatile fatty acids and MCP satisfy a major part of the dietary energy (ca. 80%) and protein (65–85%) requirements of the host (1, 2). Since the availability of fermentation products (amount and composition) impacts milk yield, milk fat, and protein synthesis, rumen fermentation is considered to be a vital process, affecting the performance of dairy animals (3). The escape of microbial cells from the rumen is followed by their digestion and absorption in the small intestine leading to the availability of amino acids, needed to satisfy the requirements of the host animal (4). Cell membranes of rumen bacteria are composed of different fatty acids like odd and branch-chain fatty acids that also contribute to fatty acid profile of milk (5).

Some phytogenic feed additives, particularly secondary plant compounds, have shown to affect the composition of the rumen microbiome, change rumen fermentation dynamics and have impact on milk production performance (6–10). So far, the majority of *in vitro* and *in vivo* studies, aimed to evaluate the use of plant secondary metabolites in ruminants, have been conducted using one or two plants or their extract or essential oils. In contrast, we wanted to test if different plant-based compounds would act synergistically and therefore decided to supplement a relatively complex mixture of phytogenic compounds derived from 10 plants with proven antioxidant or antimicrobial activity. Combinations of phytogenic antioxidants have, for example, greater potential to scavenge free radicals than individual plant compounds (11).

The plant compounds selected for this study have previously shown to be bioactive and had beneficial effects on rumen fermentation and animal performance (12). For example, supplementation of ginger improved *in vitro* fermentation characteristics by reducing ammonia nitrogen (NH_3_-N), methane and acetate to propionate ratio along with desirable effects on fibrolytic bacteria and protozoa (13). Turmeric (*Curcuma long*) possesses anti-bacterial (14), anti-parasitic (15) and antioxidant properties owing to its high content of curcumin and other curcuminoids (16). In a previous study, we highlighted the potential effects of curcumin as an epigenetic modulator with potential effects on animal physiology (17). Recently, curcumin supplementation has shown to increase milk yield and unsaturated fatty acid (oleic acid) contents of milk in dairy sheep (18). In combination with other herbs, turmeric increased fat- and energy-corrected milk yields in cows, while decreasing the acetate-to-propionate ratio in the rumen fluid (19). *Terminalia chebula* and *Phyllanthus emblica* are rich sources of tannins with potential impact on rumen fermentation; particularly a reduction of methanogenesis through decreasing rumen protozoa (20). Supplementation of *T. chebula* at 10 g per kg diet DM in sheep improved nutrient digestibility and fiber degradability possibly through increasing numbers of fibrolytic bacteria (21, 22). Inclusion of *Phyllanthus emblica* has shown to increase *in vitro* dry and organic matter degradability and the synthesis of microbial biomass, while reducing methane production (23). A combination of ajwain, fenugreek, and fennel seeds and fruits of *Terminalia chebula* and *Phyllanthus emblica*, reduced *in vitro* methane production without affecting other fermentation parameters (24). Supplementation of fenugreek led to an improvement *in vitro* dry matter degradability and *in vivo* nutrient digestion and utilization in goats (25, 26). Recently, the inclusion of licorice root has shown to increase protein and saturated fatty acid contents of milk while decreasing unsaturated fatty acids and somatic cell count of goat milk (27).

In present study, we attempted to evaluate the synergistic effects of a mixture of 10 different plant-derived compounds on the rumen bacteriome, rumen fermentation, and milk yield and composition of water buffaloes.

## Materials and Methods

### Animals, Diet, and Experimental Design

This research was carried out at Guangxi Buffalo Research Institute, Nanning, China (latitude 28° 48′N, longitude 108° 22′E). All experimental procedures used in this experiment were approved by the Ethics committee of the Chinese Academy of Agriculture Sciences, Guangxi Buffalo Research Institute, China. Twenty Murrah buffaloes of similar body weight (580 ± 25 kg), parity and stage of lactation (3–4 months) were randomly selected for this experiment and divided into four groups. The four groups of buffaloes were fed with the same basal diet supplemented with 0 (control), 15 (MP15), 25 (MP25), or 35 (MP35) g of a mixture of 10 different phytogenic substances per buffalo per d. Aside from time to exercise and swim, buffaloes were housed individually in an open-sided shed. To exercise, the buffaloes were set free in an adjacent open yard with a stocking density of 15 m^2^/buffalo. Free access to water was provided to all buffaloes throughout the day. Fans were installed in the buffalo barn to improve airflow. Buffaloes were allowed 30 min swimming time before milking. Buffaloes were machine milked twice a day. The same experimental diet consisting of maize silage, brewers' grains, and concentrate mixture was fed to all experimental buffaloes for 6 weeks. The buffalo were fed a total mixed ration (TMR) twice per day for *ad libitum* intake. The TMR was formulated to meet the dietary requirements of lactating buffalo. The respective amount of phytogenic supplement was top-dressed on TMR during morning feeding before milking and each buffalo was monitored for leftover. Details of the chemical composition of the experimental diet are given in Table 1. The first 2 weeks were considered as an adaptation period. Feed intake of individual buffaloes was measured during the last week of the experiment.

**Table 1:** Formulation and chemical composition of the basal experimental diet.

**Items**	**Content**
**Ingredient (g/kg of DM)**	
Corn silage	197
Brewers' grains	418
Concentrate feed mixture^*^	385
Total	1,000
**Chemical composition (g/kg of DM, unless otherwise stated)**	
DM (g/kg as fed)	416
OM	756
CP	158
NDF	119
ADF	81
Gross energy (kcal/kg DM)	3.41

* *Corn 17.83%; wheat bran 7.51%; Soybean meal 5.72%; Lime stone 0.5%; CaHPO_4_ 0.6%; NaHCO_3_ 0.8%; NaCl 0.7%; Premix^a^ 0.34%*.

a *The additive premix provided the following per Kg of diets: Vitamin A 550,000 IU, Vitamin E 3,000 IU, Vitamin D3 150,000 IU, 4.0 g Fe (as ferrous sulfate), 1.3 g Cu (as copper sulfate), 3.0 g Mn (as manganese sulfate), 6.0 g Zn (as zinc sulfate), 80 mg Co (as cobalt sulfate)*.

The mixed phytogenic consisted of respective parts of following plants; fennel (seeds), ajwain (seeds), ginger (tubers), *Swertia chirata* (leaves), *Citrullus colocynthis* (fruit), Turmeric, Fenugreek (seeds), *Terminalia chebula* (fruit), Licorice (roots), and *Phyllanthus emblica* (fruit). These plant parts were procured in dry, finely-ground form from Verbena Nutraceuticals Inc. (Islamabad, Pakistan). To make up the tested supplement, equal quantities of each compound were thoroughly mixed.

### Chemical Composition of the Diet and Mixed Phytogenic

Dry matter (DM), crude protein (CP), and ash content of the feed samples were analyzed according to the standard procedures (28). Neutral detergent fiber (NDF) and acid detergent fiber (ADF) were determined using an ANKOM^2000^ Fiber Analyzer (ANKOM Technology Corp., Macedon, NY, USA) including alpha-amylase and sodium sulfite (28, 29). The total polyphenolic content of mixed phytogenic was determined using the Folin-Ciocalteau's phenol reagent as reported previously (30). Gallic acid (10–60 μg/g) was used as standard. The results were expressed as mg of gallic acid equivalent (GAE) per g of MP. Total tannins were measured as tannic acid equivalent and flavonoids were determined as catechin equivalent using UV-VIS Spectrophotometer (Labomed UVD-3500 Spectro) as described previously (31). The chemical composition of the mixed phytogenic is presented in Table S1.

### Rumen Fermentation Parameters

Rumen content samples (500 ml) were collected only once, at the last day of the experiment before the morning feeding, using a stomach tube. After collection, the samples were directly transported to the laboratory. The rumen pH was measured immediately using a pH meter (HI 9024C; HANNA Instruments, Woonsocket, Rhode Island, USA). Subsequently, the rumen contents were strained through two layers of cheesecloth and subsamples were analyzed for VFA concentrations (C2, C3, C4, C5, iC4, and iC5) using a GC system (Agilent 7890A, Agilent Technologies, USA), as described by Qin (32). A sub-sample of rumen fluid (4 mL) was acidified with 4 mL of HCl (0.2 mol/L) and stored in a freezer (−20°C) for determination of NH_3_-N using the indophenols method (33). Microbial protein content was analyzed with a spectrophotometer at 595 nm using 1 mg/ml bovine serum albumin solution (Sigma-Aldrich Co., LLC, St. Louis, Missouri, USA) as standard equivalent (34).

### Milk Yield and Composition

Milk yield in the morning (at 5:00 am) and evening (at 5:00 pm) was recorded daily for each buffalo between d 15 and 42. Milk samples for determination of milk composition were collected weekly for 4 consecutive weeks. Fresh milk samples were used to analyze milk composition (milk total solids, protein, fat, and lactose) for morning and evening separately using MilkoScanTM F120 (FOSS, Hillerød, Denmark). Energy corrected milk (ECM) was calculated according to Tyrrell and Reid (35):

$$\begin{array}{l} \text{ECM}=0.327\times \text{Milk yield }\left(\text{kg}\right)+12.95\times \text{Fat yield }\left(\text{kg}\right)+7.20\\ \;\times \text{Protein }\left(\text{kg}\right). \end{array}$$

### Determination of Fatty Acid Profile in Milk

Samples from morning and evening milking were pooled (relative to the quantity of milk produced) for each week separately. Milk samples from each week were stored at −20°C until analysis of fatty acids. Briefly, 20 mL of milk was centrifuged in a 50 mL falcon tube at 17,800 × g for 30 min at 4°C. After centrifugation, the above fat layer (1.0 g) was transferred to a 1.5 mL Eppendorf tube and left at room temperature (~20°C) for ~20 min to allow fat to melt. After that, it was centrifuged at 19,300 × g for 20 min at room temperature in a microcentrifuge. Centrifugation of fat separated the sample into 3 layers: top layer containing lipid; middle layer containing protein, fat, and other water-insoluble solids; and bottom aqueous layer (36). Milk fatty acids were trans-esterified with sodium methoxide according to Zahran and Tawfeuk (37). Briefly, 2.0 mL of n-hexane were added to 40 ul of butterfat and vortexed for 30 s followed by the addition of 2 mL of sodium methoxide (0.4 mol). After vortexing, the mixture was allowed to settle for 15 min. The upper phase, containing the fatty acid methyl ester (FAME), was recovered and analyzed by an Agilent 7890B Gas chromatography (GC-FID) with a polar capillary column CP-Sil^®^-88 100 m, 0.25 mm id, 0.2 μm film thickness (Agilent Technologies, USA). Helium was used as a carrier gas at a flow rate of 20 cm s^−1^ and split ratio 100:1. The column temperature profile was held at 100°C for 5 min, ramp to 240°C @ 4°C min^−1^; hold at 240°C for 30 min. A sample volume of 1.0 μL was injected. The FAME was identified by comparing their relative and absolute retention times with FAME standards (from C4:0 to C22:0). Fatty acid contents are presented as percentage of total fat weight (wt%/wt%).

### DNA Extraction and Sequencing of the 16S rRNA Gene

The DNA was extracted from 1 mL of frozen rumen content (both solid and fluid phase) using the CTAB bead-beating method (38). The quality of DNA was checked using a spectrophotometer (NanoDrop2000, Thermo Scientific, USA). High throughput (Illumina MiSeq) sequencing of the 16S rRNA gene was carried out using barcoded primers for V3–V4 region (39). DNA libraries were sequenced using a 2 × 300 paired-end sequencing module (Illumina, San Diego). Resultant paired-end sequence reads were joined together using their overlap relationship (minimum 10 bp) allowing maximum mismatch ratio of 0.2 using FLASH and Trimmomatic software. After pruning, optimized sequence reads were aligned against the SILVA database, Release128 (http://www.arb-silva.de) for identification of Operational Taxonomic Units (OTU) using cluster identity threshold of 97% (40, 41). After that taxonomy of each sequence (OTU representative) was analyzed by RDP Classifier (http://rdp.cme.msu.edu/) against the database (confidence threshold of 0.7). Taxonomic assignment of rumen bacteria was performed with bioinformatics pipeline of Qiime software (http://qiime.org/scripts/assign_taxonomy.html) as described previously (42).

The bacterial diversity of treatment groups was determined by analyzing alpha and beta diversity indices. Population richness (Chao, ACE) and evenness (Shannoneven and Simpsoneven) of rumen bacteria were analyzed for each sample (43). Alpha diversity was estimated by determining Shannon and Simpson indices (44–47). Beta diversity index was calculated to analyze rumen bacterial diversity across different treatment groups using Bray-Curtis dissimilarities (48). Bray-Curtis dissimilarities among different treatment groups were evaluated non-parametrically by utilizing permutation analysis of variance method (PERMANOVA using 999 permutations) as previously reported (49). Redundancy analysis (RDA) was performed at the bacterial genus level using VFAs and milk yield parameters as explanatory variables in the vegan R package (version 3.2).

### Statistical Analysis

Effect of MP on all parameters related to milk yield and composition; DM intake, rumen fermentation, and bacterial alpha diversity were analyzed using the general linear model in SAS (SAS Institute Inc., Cary, NC, USA) with treatment as a fixed effect and buffalo as a random effect nested in treatment group. The Duncan's multiple range test was used as a *post-hoc* test to identify differences among treatment groups. We also analyzed three orthogonal contrasts including all MP treatments vs. the control, linear effect of MP dose, and quadratic effect of MP dose. Treatment effects were declared significant at *P* < 0.05 and trends were discussed at 0.05 ≤ *P* < 0.1. The abundances of bacterial phyla and genera were compared using the Kruskal-Wallis H test with a false discovery rate (FDR) correction and Scheffer as a *post-hoc* test to elucidate differences across treatment groups.

Spearman's rank correlation (r) analyses were performed with the vegan R package (version 3.2) to analyze the association of relative abundance of bacterial genera with rumen fermentation and milk yield parameters. Correlation heatmaps were constructed using the corrplot R package. In the two-dimensional heat map, change in defined color and its depth indicates the nature and strength of the correlation, respectively. Asterisk sign was used when the r value was >0.1 and the *P*-values were < 0.05 (^*^0.01 < *P* ≤ 0.05, ^**^0.001 < *P* ≤ 0.01, ^***^P ≤ 0.001).

## Results

### Rumen Fermentation Parameters

Supplementation of MP increased ruminal pH (*P* = 0.029) in MP15 and MP35 but no change in pH was observed in MP25 compared to the control (Table 2). There was no effect of treatment on any other rumen fermentation parameter.

**Table 2:** Effect of mixed phytogenic on rumen fermentation parameters.

**Item**	**Treatments**				**SEM**	***P*-value**
	**Control**	**MP15**	**MP25**	**MP35**		
pH	6.68^b^	6.88^a^	6.79^ab^	6.81^a^	0.026	0.029
TVFAs (mmol/L)	34.87	31.07	31.34	32.87	1.027	0.586
Acetate (mmol/L)	17.54	16.07	16.50	16.95	0.446	0.771
Propionate (mmol/L)	9.99	8.43	8.52	8.99	0.353	0.408
Isobutyrate (mmol/L)	0.63	0.69	0.63	0.65	0.017	0.714
Butyrate (mmol/L)	5.55	4.79	4.69	5.20	0.240	0.611
Isovalerate (mmol/L)	0.68	0.69	0.61	0.67	0.036	0.884
Valerate (mmol/L)	0.45	0.39	0.37	0.40	0.025	0.699
Acetate/Propionate	1.78	1.91	1.93	1.89	0.039	0.559
MCP (mg/mL)	37.93	42.23	43.72	38.73	1.418	0.436
NH_3_-N (mg/mL)	11.94	10.66	9.91	10.24	0.837	0.857

*MP15, mixed phytogenic fed @ 15 g/d/head; MP25, mixed phytogenic fed @ 25 g/d/head; MP35, mixed phytogenic fed @ 35 g/d/head; Control, without mixed phytogenic; TVFAs, Total volatile fatty acids; MCP, Microbial crude protein; NH_3_-N, Ammonia Nitrogen*.

a,b *Values with different superscripts in the same row differ significantly (P < 0.05)*.

### Dry Matter Intake (DMI), Milk Yield, and Composition

There was no treatment effect on DMI and milk production performance (Table 3).

**Table 3:** Effect of mixed phytogenic on milk yield parameters.

**Parameter**	**Control**	**MP15**	**MP25**	**MP35**	**SEM**	***P*-value**
Dry matter intake (kg/d)	7.78	8.16	8.06	8.26	0.109	0.479
Milk yield (kg/d)	8.69	8.52	8.50	8.57	0.554	1.000
Fat corrected milk (kg/d)	13.36	14.03	13.07	14.61	0.740	0.907
Energy corrected milk (kg/d)	14.45	15.04	14.01	15.49	0.805	0.937
Protein (%)	4.99	5.00	4.65	4.89	0.064	0.181
Protein yield (kg/d)	0.44	0.43	0.39	0.42	0.028	0.965
Fat (%)	7.83	8.28	7.73	8.69	0.248	0.539
Fat yield (kg/d)	0.65	0.71	0.64	0.74	0.034	0.339
Total solids (%)	19.25	19.56	18.70	19.84	0.280	0.562
Solid not fat (%)	10.70	10.53	10.32	10.33	0.072	0.194
Lactose (%)	5.32	5.21	5.42	5.21	0.037	0.108

*MP15, mixed phytogenic fed @ 15 g/d/head; MP25, mixed phytogenic fed @ 25 g/d/head; MP35, mixed phytogenic fed @ 35 g/d/head; Control, without mixed phytogenic*.

### Fatty Acids Composition of Milk

Supplementation of MP increased (*P* < 0.05) short-chain fatty acids in MP15 compared to other groups (Table 4). Myristic acid (C14:0) tended to increase, while C18:0 tended to decrease in MP15 as compared to other groups. The C16:1 content and its unsaturation index was higher (*P* < 0.05) in MP35 as compared to the control and other treatment groups. Furthermore, C18:3n3 was (*P* < 0.05) higher in control, MP15 and MP25, as compared to MP35. There was no treatment effect on total UFA, MUFA, and PUFA content as well as omega6 to omega3 ratio.

**Table 4:** Milk fatty acids profile across different treatment groups.

**Fatty acid**	**Common name**	**Control**	**MP15**	**MP25**	**MP35**	**SEM**	***P*-value**
C4:0	Butyric acid	0.86^ab^	0.95^a^	0.83^bc^	0.74^c^	0.035	0.001
C6:0	Caproic acid	1.03^ab^	1.12^a^	0.96^b^	1.05^ab^	0.019	0.023
C8:0	Caprylic acid	0.68^ab^	0.78^a^	0.62^b^	0.73^a^	0.018	0.014
C10:0	Capric acid	1.49^b^	1.88^a^	1.39^b^	1.64^ab^	0.053	0.014
C12:0	Lauric acid	2.19^b^	2.63^a^	2.03^b^	2.32^ab^	0.066	0.016
C14:0	Myristic acid	10.64^b^	11.55^a^	10.45^b^	11.03^ab^	0.161	0.054
C14:1	Myristoleic acid	1.04	1.11	1.10	1.14	0.016	0.125
C16:0	Palmitic acid	31.91	32.22	32.76	33.19	0.366	0.671
C16:1	Palmitoleic acid	1.88^b^	1.77^b^	1.90^b^	2.19^a^	0.044	0.001
C17:0	Margaric acid	0.3	0.32	0.30	0.28	0.009	0.474
C18:0	Stearic acid	15.97^a^	14.50^b^	15.41^ab^	14.63^ab^	0.267	0.086
C18:1	Oleic acid	28.87	27.69	28.89	27.78	0.354	0.488
C18:2n6	Linoleic acid	1.42	1.56	1.53	1.43	0.029	0.255
C18:3n3	α-Linolenic acid	0.43^ab^	0.48^a^	0.47^a^	0.39^b^	0.014	0.035
C18:3	Linolenic acid	1.51	1.40	1.47	1.51	0.042	0.639
**Group of fatty acids, g/100 g of fatty acids**							
SFA		64.82	65.97	64.65	65.53	0.393	0.617
UFA		35.17	34.02	35.34	34.46	0.393	0.617
MUFA		31.81	30.59	31.89	31.12	0.357	0.554
PUFA		3.36	3.43	3.45	3.34	0.060	0.891
SCFA		3.98^b^	4.75^a^	3.71^b^	4.12^b^	0.097	0.001
MCFA		47.51	49.31	48.26	49.83	0.504	0.365
LCFA		48.51	45.94	48.03	46.05	0.553	0.224
n-6/n-3		3.35	3.47	3.43	3.86	0.128	0.419
**Unsaturation index, %**							
C14:1/(C14:0 + C14:1)		9.19	8.84	9.63	9.42	0.144	0.283
C16:1/(C16:0 + C16:1)		5.61^b^	5.25^b^	5.49^b^	6.25^a^	0.125	0.01
C18:1/(C18:0 + C18:1)		64.41	65.9	65.18	65.44	0.387	0.427

*SFA, Saturated fatty acids; UFA, unsaturated fatty acids; MUFA, monounsaturated fatty acids; PUFA, poly unsaturated fatty acids; SCFA, short-chain fatty acids; MCFA, medium-chain fatty acids; LCFA, long-chain fatty acids; SCFA included the C4:0, C6:0, C8:0, and C10:0 fatty acids; MCFA included all linear fatty acids from C12:0 to C16:1; LCFA included all linear fatty acids from C17:0 to C18:3. MP15, mixed phytogenic fed @ 15 g/d/head; MP25, mixed phytogenic fed @ 25 g/d/head; MP35, mixed phytogenic fed @ 35 g/d/head; Control, without mixed phytogenic*.

a,b,c *Values with different superscripts in the same row differ significantly (P < 0.05)*.

### Rumen Bacterial Diversity

**Figure 1:**
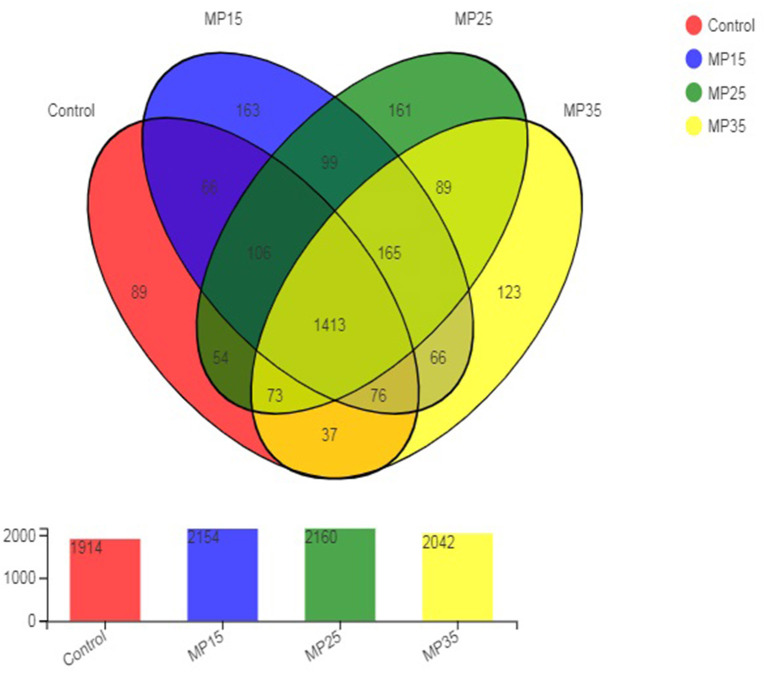
Distribution of OTU across different treatment groups.

High throughput sequencing of the 16S rRNA gene revealed a total of 2,780 OTU in the rumen content samples. The distribution of shared and unique OTU of the four treatment groups is presented in Figure 1. The highest number of OTU was detected in buffaloes supplemented with MP25, compared to control and other groups. The number of OTU increased in response to MP15 and MP25 but decreased for MP35 as compared to the control. A total of 1,413 OTU were shared by all groups, while the number of unique OTU was 536. The highest number of unique OTU was found in MP15 (163) followed by MP25 (161), MP35 (123), and the control (50).

**Figure 2:**
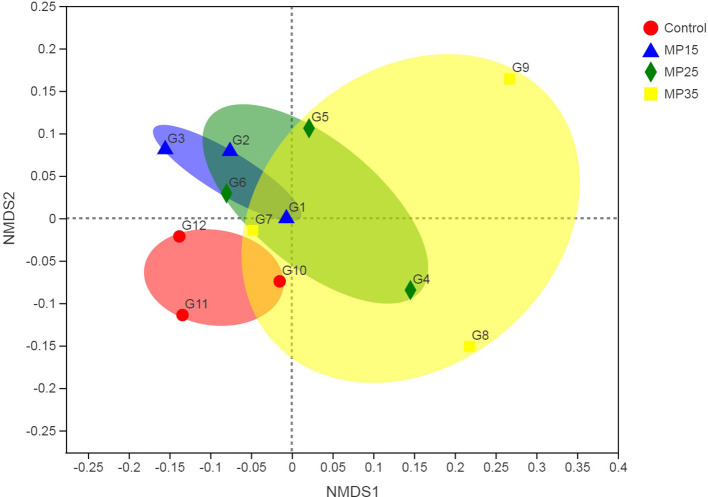
First two dimensions from the (non-metric) multi-dimensional scaling of the Bray-Curtis dissimilarity matrix. Samples were grouped by feed additive. PERMANOVA amongst all groups *p* = 0.025 (using 999 permutations).

Treatment had no effects on alpha diversity parameters (Table 5). Analysis of beta diversity showed the difference between groups caused by dietary treatment. The first two dimensions from the (non-metric) multi-dimensional scaling (NDMS) of the Bray-Curtis dissimilarity matrix are presented in Figure 2. Samples were grouped by the level of MP and PERMANOVA (using 999 permutations) amongst all groups showed effect of treatment (*P* = 0.025).

**Table 5:** Effect of mixed phytogenic on bacterial alpha diversity parameters.

**Items**	**Control**	**MP15**	**MP25**	**MP35**	***P*-value**
Shannon	5.619	5.759	5.854	5.908	0.095
Simpson	0.013	0.012	0.010	0.008	0.231
ace	1892.8	1999.3	1956.7	1930.4	0.620
Chao	1777.1	2001.4	1955.7	1978.1	0.102
Shannoneven	0.785	0.787	0.800	0.814	0.260
Simpsoneven	0.063	0.057	0.069	0.093	0.451

*MP15, mixed phytogenicfed @ 15 g/d/head; MP25, mixed phytogenic fed @ 25 g/d/head; MP35, mixed phytogenicfed @ 35 g/d/head; Control, without mixed phytogenic*.

### Relative Abundance of Rumen Bacteria

**Figure 3:**
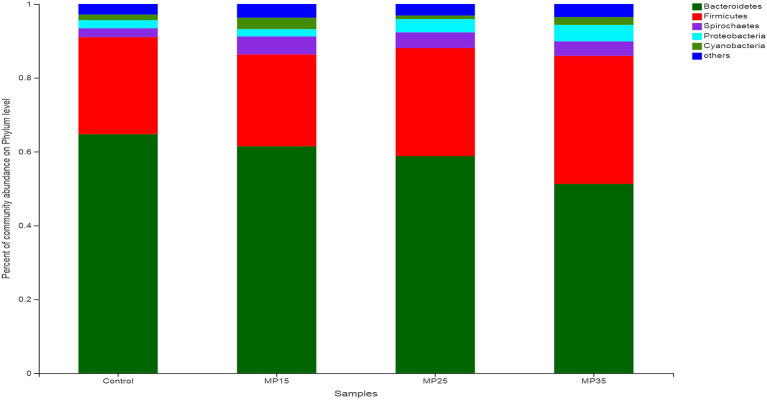
Relative abundance of bacterial phyla across treatment groups.

*Bacteroidetes* and *Firmicutes* were the most dominant phyla representing between 85 and 91% of total bacteria detected in the rumen of the buffaloes (Figure 3). The relative abundance of *Firmicutes* and *Proteobacteria* increased while *Bacteroidetes* and *Spirochaetes* decreased numerically in response to treatment compared to the control (Table S2). The abundance of *Cyanobacteria* increased in MP15 and MP35 but decreased numerically in MP25 as compared to the control group.

**Figure 4:**
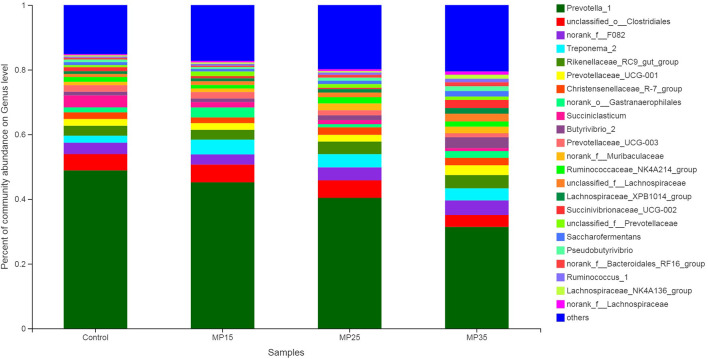
Relative abundance of bacterial genera across treatment groups.

*Prevotella* was the dominant genus in all four treatments, representing about 31–49% of all sequences (Table S3). Relative abundance of *Prevotella* decreased numerically with increasing levels of MP (Figure 4). Second most abundant genus was *o-Clostridales*, which increased with supplementation particularly in MP15 (5.74%) and MP25 (5.53%) as compared to MP35 (3.62%) and control (4.96%). Abundance of *f__F082* increased in MP25 (4.01%) and MP35 (4.40%) compared to MP15 (3.09%) and control (3.45%). Similarly, abundance of *Rikenellaceae_RC9_gut*_group also increased in MP25 (3.95%) and MP35 (4.01%) compared to MP15 (2.99%) and the control (3.09%). *Treponema* decreased linearly in response to MP supplementation. Highest relative abundance of *Christensenellaceae_R-7_*group was observed in MP25 (2.39%) and MP35 (2.33%) as compared to MP15 (1.80%) and control group (2.07%). Reduced abundance of *Succiniclasticum* and *Prevotellaceae_UCG-003* was observed in MP-supplemented buffaloes compared to the control. Relative abundance of *Butyrivibrio* increased with supplementation of MP, particularly in MP35, compared to the control. The relative abundance of *Ruminococcaceae* also increased as result of MP supplementation. *Pseudobutyrivibrio* decreased in response to MP15 but was present in greater abundance in MP25 and MP35 compared to the control. The relative abundance of *Succinibrionaceae_UCG-002* increased in MP35 (2.33%) compared to MP15 (0.66%), MP25 (0.41%), and the control (1.17%).

### Association of Bacteria With Rumen Fermentation Parameters

**Figure 5:**
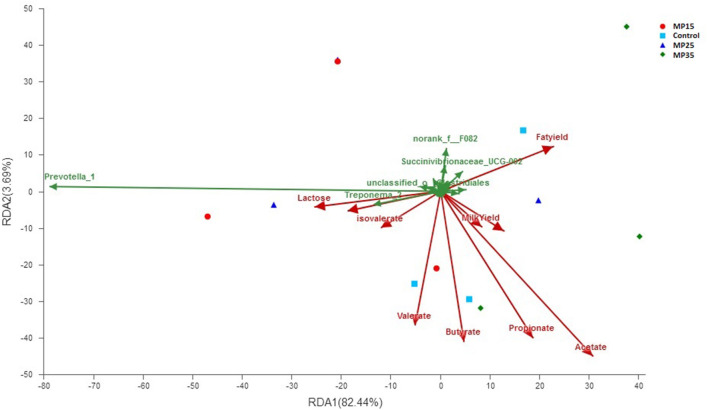
Biplot of RDA analysis on genus level between bacterial genera, VFA, and milk yield parameters.

Redundancy analysis showed acetate contributed to the bacterial community differences at genus level (contribution = 56.8%, *P* = 0.023) among the four treatment groups (Figure 5). Milk yield and composition parameters did not contributed to overall differences in bacterial genera. Two bacterial genera *Prevotella_1* (contribution = 78.6, *P* = 0.04) and *Treponema_2* (contribution = 13.3, *P* = 0.045) contributed substantially to the compositional differences in rumen bacteriome (Figure 5).

**Figure 6:**
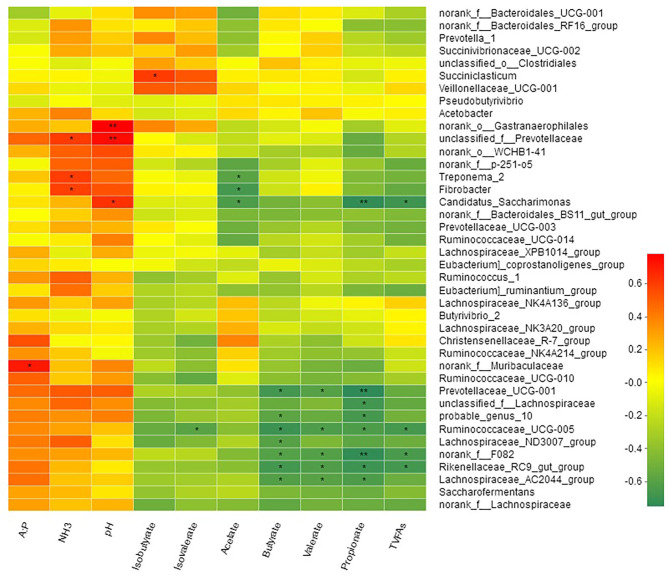
Correlation of bacterial genera with rumen fermentation parameters.

Spearman's correlation between the relative abundance of bacterial genera and rumen fermentation parameters is shown in Figure 6. Acetate concentrations were negatively correlated with *Treponema_2* (*R* = −0.59; *P* < 0.05), *Fibrobacter* (*R* = −0.66; *P* < 0.05), and *Candidatus_Saccharimonas* (*R* = −0.64; *P* < 0.05). Propionate, butyrate and valerate were negatively correlated with genus *f_F082, Rikenellaceae_RC9_gut_group, Prevotellaceae_UCG-001* (*R* = −0.75, *P* < 0.01, *Lachnospiraceae_AC2044_group, Ruminococcaceae_UCG-005, Lachnospiraceae_ND3007_group*, and *probable_genus_10*, while two genera *Ruminococcaceae_NK4A214_group* and *Candidatus_Saccharimonas* were negatively correlated with propionate but not with butyrate (Table S4). Isobutyrate showed a positive correlation (*R* = 0.60; *P* < 0.05) with genus *Succiniclasticum*, while isovalerate was negatively correlated (*R* = −0.59; *P* < 0.05) with *Ruminococcaceae_UCG-005*. Only one bacterial genus *f_Muribaculaceae* showed positive correlation (*R* = 0.71; *P* < 0.05) with acetate to propionate ratio. TVFAs showed a negative correlation with genus *f_F082, Rikenellaceae_RC9_gut_group, Ruminococcaceae_UCG-005*, and *Candidatus_Saccharimonas*. Three bacterial genera *Rikenellaceae_RC9_gut_group, f_Prevotellaceae*, and *Fibrobacter* were positively correlated with the ruminal concentration of NH_3_-N.

### Association of Rumen Bacteria With DMI, Milk Yield, and Composition

**Figure 7:**
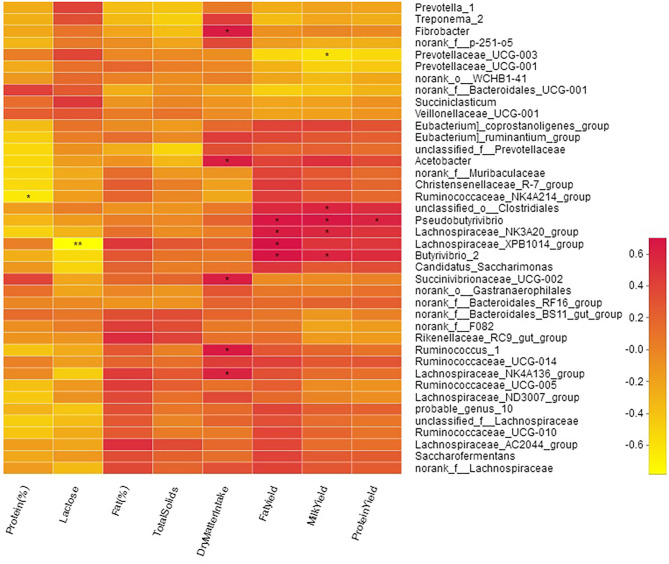
Correlation of bacterial genera with milk yield parameters.

Milk yield was positively correlated with genera *o_Clostridiales* (*R* = 0.59; *P* < 0.05), *Butyrivibrio_2* (*R* = 0.59; *P* < 0.05), *Pseudobutyrivibrio* (*R* = 0.67; *P* < 0.05), and *Lachnospiraceae_NK3A20_group* (*R* = 0.58; *P* < 0.05; Figure 7, Table S5). A moderate negative (*R* = −0.61; *P* < 0.01) correlation of milk yield was observed with *Prevotellaceae_UCG-003*. Milk protein contents were negatively (*R* = −0.64; *P* < 0.05) correlated with *Ruminococcaceae_NK4A214_group* while milk protein yield was positively correlated (*R* = 0.61; *P* < 0.05) with *Pseudobutyrivibrio*. Milk fat yield showed positive correlation with; *o_Clostridiales* (*R* = 0.56; *P* < 0.05), *Butyrivibrio_2* (*R* = 0.69; *P* < 0.05), *Lachnospiraceae_XPB1014_group* (*R* = 0.70; *P* < 0.05), *Pseudobutyrivibrio* (*R* = 0.68; *P* < 0.05), and *Lachnospiraceae_NK3A20_group* (*R* = 0.64; *P* < 0.05). Milk lactose was negatively (*R* = −0.78) correlated with *Lachnospiraceae_XPB1014_group*. Bacteria specialized in fiber and polysaccharides degradation including *Succinivibrionaceae_UCG-002* (*R* = 0.63; *P* < 0.05), *Ruminococcus_1* (*R* = 0.65; *P* < 0.05), *Lachnospiraceae_NK4A136_group* (*R* = 0.60; *P* < 0.05), *Fibrobacter* (*R* = 0.65; *P* < 0.05), and *Acetobacter* (*R* = 0.62; *P* < 0.05) were positively correlated with DMI (Figure 7).

## Discussion

### Rumen Fermentation Parameters

The mixed phytogenic tested in this study had no effect on rumen fermentation parameters of buffaloes except pH. An increase in rumen pH in response to supplementation of phytochemicals (flavonoids and polyphenols) in ruminants has been reported earlier (51, 52). A stabilization of rumen pH and prevention of acidotic bouts would be particularly beneficial for ruminants fed large amount of readily fermentable carbohydrates which can decrease rumen pH rapidly and alter fermentation kinetics and the composition of rumen microbiome. Strong declines in rumen pH reduce the activity of cellulolytic bacteria (53), shift bacterial populations and promote lysis of gram-negative bacteria leading to an increase in lipopolysaccharides (LPS) in the rumen (54, 55). Plant polyphenols have shown to increase rumen pH and stimulate the diversity of rumen microbiota, which is commonly high in conditions of physiological rumen pH and regular rumen function (56).

Rumen pH plays a crucial role in fiber degradation as it directly affects bacterial adhesion to cellulosic material (57, 58). This can lead to a reduction in fiber digestion, as frequently observed in animals fed high-grain diets (59). In addition, fibrolytic bacteria like *Rumiococcus* and *F. Succinogenes* are highly sensitive to even mildly acidic pH (60). Our findings indicate that the tested combination of phytogenics might improve the performance and health of ruminants by preventing excessive drop in pH and subsequent accumulation of LPS in rumen. However, it needs to be emphasized that we measured rumen pH only once as spot sampling just before the morning feeding. It may not be reflective of pH changes over the course of the day, which are important variables to consider. Earlier studies have reported the strong diurnal variation of rumen fermentation parameters and particularly pH (61). The limited number of buffaloes enrolled in the experiment and the fact that we only sampled each buffalo once, is a limitation of our study. In addition, we also did not collect rumen samples at the beginning of the experiment but considered the control group as baseline for our experiment. A more extensive rumen sampling protocol should be followed in further experiments.

We observed an increase in the relative abundance of well-known bacterial genera like *Pseudobutyrivibrio, Butyrivibrio*, and *Succinivibrioanceae*. These bacteria form butyrate and propionate which can subsequently affect milk composition especially the milk fat content. The shift in the relative abundance of certain bacterial genera had, besides the discussed modulation of rumen pH, no other effect on rumen fermentation. This might be due to the functional redundancy of the rumen microbiota. The rumen microbiome possesses the ability to adapt to long term exposure to inhibitory substances, like some phytogenics but the effectiveness of the adaptation is dependent on the robustness and diversity of the microbiome, length of exposure, and the concentration of the inhibitor (62). No change in rumen fermentation parameters in response to phytogenic compounds like peppermint oil, garlic, and *Piper sarmentosum* was reported earlier (63, 64).

### DMI, Milk Yield, and Composition

Earlier studies have also reported no effect of plant compounds, such as propolis polyphenols, garlic and peppermint on DMI and the apparent digestibility of nutrients in buffaloes (63, 65). As it was the case in the present study, other studies also reported that polyphenolic compounds had no negative impact on milk yield. For example, supplementation of propolis polyphenols had no effect on milk yield and concentration of milk solids in dairy cows (66). Studies using a blend of different phytochemicals like cinnamaldehyde, eugenol and capsicum also reported no effects on milk yield in dairy cattle (67–69).

### Milk Fatty Acid Contents

The major milk fatty acids were C16:0 and C18:1, followed by C18:0 and C14:0, which is in agreement with earlier studies in dairy cattle (70, 71). Contents of SFA (65%) and UFA (35%) measured in our study are similar to earlier reports in cattle and buffaloes (72, 73).

Supplementation of MP15 increased the content of short-chain fatty acids (C4 to C10:0) in milk. The increase in C18:3n3 in response to MP15 and MP25 means that MP has the potential to affect de novo synthesis of fatty acids. The tendency to decrease the percentage of stearic acid (C18:0), a major saturated fatty acid, is desirable from a human health point of view. Polyphenolic compounds have shown to affect microbial biohydrogenation by inhibiting specific rumen bacteria, this can lead to a more desirable fatty acid composition of milk (74–76). Condensed tannins have shown to partially inhibit the last step of C18:3 biohydrogenation in the RUSITEC system (77). Durmic et al. (78) reported that tannins extracted from *Acacia mearnsii* inhibited *Clostridium proteoclasticum* but exhibited no effect on *Butyrivibrio fibrisolven*, revealing selective inhibition of rumen bacteria involved in biohydrogenation.

Earlier studies reported that polyphenolic-rich forage increased the α-linoleic acid content of milk in sheep (79, 80). Higher abundance of *Butyrivibrio* and *Pseudobutyrivibrio* was associated with an increase in the content of unsaturated fatty acids owing to their positive correlation with linoleic acid and n-3 fatty acid content of milk (81). The decrease in stearic acid (C18:0) together with the increase in n-3 fatty acid contents, is in agreement with earlier studies that reported similar findings in response to supplementation of tannins in dairy sheep (82). Based on the ratio of C14:1 to C14:0 (a proxy of desaturation), it has been suggested that tannins can enhance the activity of stearoyl Co-A desaturase enzyme (SCD), which mediates the conversion of stearic acid to oleic acid and vaccenic acid to conjugated linolenic acid (CLA). In particular, SCD has shown to contribute almost 50% of oleic acid and cis-9, trans-11 CLA secreted in sheep milk (83). This implies that tannins can increase milk unsaturated fatty acids especially n-3 fatty acids not only by mediating rumen biohydrogenation but also through enhancing SCD activity (82, 84, 85).

Since we did not determine the fatty acids content of the rumen microbial biomass, we are unable to associate microbial abundance with the fatty acid profile in milk. This should be attempted in future studies.

### Rumen Bacterial Diversity

Supplementation of MP had no effect on bacterial alpha diversity. However, beta diversity was impacted by MP. Similar results regarding alpha and beta diversity have been reported earlier in response to grape-pomace which is rich in polyphenols (86).

As it was the case in this study, *Bacteroidetes* and *Firmicutes* are the major bacterial phyla in both dairy cattle and buffaloes (50, 87–90). A linear increase in *Firmicutes* was observed together with a decrease in *Bacteroidetes*. The highest increase in relative abundance in *Firmicutes* was observed in response to the highest dose of phytogenics (MP35) and resulted in a corresponding decrease in *Bacteroidetes*. An increase in *Firmicutes-to-Bacteroidetes* ratio in response to supplementation of plant flavonoids has been reported earlier (50). A major function of rumen *Bacteroidetes* is the breakdown of polysaccharide, along with various other activities (91). *Firmicutes* are particle-associated bacteria, which produce butyrate. Numerically higher concentration of butyrate in MP35 was associated with a greater abundance of *Firmicutes*. Furthermore, buffaloes in this group also had higher milk fat percentage and fat yield, likely due to the positive association of *Firmicutes-to-Bacteroidetes* ratio and milk fat yield, as previously reported (92).

In the present study, *Prevotella* was the dominant genus across all treatment groups. This is in agreement with earlier studies in buffalo (93–95). Supplementation of MP linearly decreased the abundance of *Prevotella*, with the greatest reduction (1.6-fold) in MP35 compared to the control. The decrease in *Prevotella* was correlated with numerically higher DMI, fat corrected milk (FCM), ECM, fat (%), and milk fat yield in MP35 as compared to the control, most likely due to the negative association of *Prevotella* with DMI and milk fat content (92, 96). We also observed a negative correlation of *Prevotella* with acetate, propionate, milk yield and fat (%) but these correlations were weak (*R* = 0.2–0.37) and not significant. In contrast, earlier studies have also reported a positive correlation of *Prevotella* with acetate and butyrate in dairy cows (97, 98) and butyrate in buffaloes (99). *Prevotella* species are more specialized in protein degradation, peptide fermentation and their uptake in the rumen (100). Similar to *Prevotella*, lower abundance of *Succiniclasticum* and *Prevotellaceae*_UCG-003 was observed in buffaloes supplemented with MP compared to the control. In response to MP35, we detected a 3-fold increase in the relative abundance of *Butyrivibrio*, together with a 1.8-fold increase in *Pseudobutyrivibrio* compared to the control. Bacterial taxa like *Firmicutes, Butyrivibrio*, and *Pseudobutyrivibrio*, are important degraders of polysaccharides in the rumen and produce formate, butyrate, and acetate (101).

Plant phenolic compounds like thymol; have shown to increase the relative abundance of *Firmicutes in vitro* (up to 82.8%) mainly by inhibiting more sensitive non-*Firmicutes* (*Bacteroidetes*) bacteria (102). In contrast, plant essentials oils extracted from *Origanum vulgare*, garlic and peppermint have shown to decrease the abundance of *Firmicutes* and methane production, while increasing *Bacteroidetes* (7). The increase in *Proteobacteria* in response to MP supplementation was interesting as a substantial increase (2-fold) in the relative abundance of *Succinibrionaceae* in response to MP35 was also observed. Previously, plant secondary metabolites (8-hydroxyquinoline, α-terpineol, camphor, bornyl acetate, α-pinene, thymoquinone, and thymol) have shown to increase the relative abundance of *Succinibrionaceae* (102). In study *Succinibrionaceae* was a dominant family of *Proteobacteria*, which is in agreement with earlier data from cattle (103). The major fermentation product of this bacterial family is succinate which is subsequently converted to propionate in the rumen, so it creates the possibility of competition between *Succinivibrioanceae* and methanogens to utilize hydrogen as a substrate to produce succinate and propionate instead of methane. In line with this, greater abundance of *Succinivibrioanceae* was negatively correlated with methane production (*R* = −0.72) in cattle (102). Substantially higher abundance of *Succinivibrioanceae* has been observed in beef cattle with low methane production compared to cattle with higher emissions (103). In addition to the fact that methane is a strong greenhouse gas, reduced losses of methane can also be associated with an improvement in feed efficiency in ruminants. Since we did not measure methane production or total methanogens, we can only speculate about the effect of MP on methane emissions. However, the detected shift in rumen bacteriome toward more beneficial bacteria like *Pseudobutyrivibrio, Butyrivibrio* and *Succinivibrioanceae* make it somewhat likely that the tested phytogenic has not only positive impact on production performance but also greenhouse gas intensity of milk.

### Association of Bacteria With Rumen Fermentation and Milk Yield Parameters

The Spearman's correlation analysis revealed 28 negative and 8 positive correlations of bacterial genera with rumen fermentation parameters. Three bacterial genera *Fibrobactor, Treponema_2*, and *f_Prevotellaceae* had a modest positive correlation with ruminal NH_3_-N concentrations. *Treponema* belongs to phylum *Spirochetes* which mostly ferments soluble sugars to formic acid, acetic acid, lactic acid, and succinic acid (104). *Succiniclasticum* was positively correlated with the concentration of isobutyrate in the rumen as this genus of bacteria is associated with the formation of succinate from starch degradation leading to the subsequent production of *propionate* (105). Moreover, increased abundance of *Succiniclasticum* in high-producing dairy cows has been associated with greater propionate production (106). In our study, we observed very few positive correlations in contrary to earlier studies reporting various strong correlations of bacterial genera with VFA in the rumen of dairy cows (81, 107) and buffaloes (99). This may be attributed to the low variation observed in fermentation parameters, which was likely due to the relatively low sample size and the fact that we only sampled once instead of multiple times over the course of the day. We took rumen samples once from each buffalo using the stomach tube at the end of the experiment; consequently we had a total of five samples per treatment. The relatively low number of buffaloes per treatment and only one rumen sampling are the main limitations of the present study. To evaluate potential effects of MP on rumen fermentation and shifts in the bacterial population in more detail, further studies are required involving a larger cohort of animals and multiple rumen samplings.

*Fibrobactor* is one of the most active cellulolytic bacteria which ferment only cellulose, glucose, and cellobiose, its primary end products are acetic and succinic acid (104). Unsurprisingly, in this study presence of Fibrobactor was positively correlated with DMI due to its ability to breakdown fiber. However, the negative correlation between *Fibrobactor* and acetate is difficult to explain. All five bacterial genera (*Succinivibrio, Ruminococcus, Lachnospiraceae, Fibrobacter, and Acetobacter*) which were positively correlated with DMI are well-known cellulolytic and amylolytic bacteria (108). Dry matter intake has a direct association with milk production so the relationship of these bacteria with DMI, as observed in this study, indicates their potential in enhancing milk yield in buffaloes (109).

Our study showed a positive correlation of *Pseudobutyrivibrio* with milk, fat and protein yield. The positive impact of *Pseudobutyrivibrio* on milk yield parameters has been reported earlier in dairy cows (108). A positive correlation of *Butyrivibrio* and *Lachnospiraceae* with milk yield and protein has also been reported (93). Furthermore, a positive correlation of *Butyrivibrio* with average milk fat, milk solid, and total milk yield has been reported in buffaloes (99). *Butyrivibrio* and *Pseudobutyrivibrio* ferment structural carbohydrates (hemicellulose, xylan, and pectin) to butyrate (110). However, a negative correlation of *Butyrivibrio* species with milk fat yield has also been reported in dairy cattle (81, 96). The substantial increase in the relative abundance of *Butyrivibrio* (3-fold) and *Pseudobutyrivibrio* (1.8-fold) in this study was correlated with numerically higher milk fat (%), DMI, FCM, ECM, and DMI in buffaloes supplemented with MP35. An unclassified genus of family *Prevotellaceae* showed a negative correlation with milk yield which is also in agreement with earlier reports revealing a negative association of *Prevotella* with DMI and milk fat content (92, 96). One unclassified genus belonging to *Clostridiales* showed a positive correlation with milk yield in the present study. Previous reports have shown substantial differences in abundance of these taxa in beef steers with high and low residual feed intake (111).

Our study found that the tested mixed-phytogenic has the potential to stabilize rumen pH which may be beneficial especially for ruminants in intensive grain-based feeding systems. An increase in *Firmicutes-to-Bacteroidetes* ratio in response to mixed phytogenic substances reveals their synergistic potential to increase milk fat yield in buffaloes. The significant increase in omega-3 and numeric increase in PUFA in response to MP15 and MP25 may be beneficial from a human health perspective. Our study provides new information regarding the potential effect of a mixed phytogenic on the rumen microbial population, particularly rumen bacteria, and their potential association with fermentation and milk performance parameters. The use of tested mixture of different phytogenics could lead to improvements in production performance and digestive health of buffaloes. However, further studies on larger cohorts are required to solidify these first results and explore the shift in the rumen bacteriome and their impact on production related traits in depth.

## Conclusions

Supplementation of MP increased rumen pH and n-3 fatty acid content of milk, while decreasing its stearic acid content. Additionally, MP promoted bacteria that are positively associated with milk and fat yield (*Firmicutes-to-Bacteroidetes ratio, Pseudobutyrivibrio, Butyrivibrio*, and *Succinivibrioanceae*). Overall, our findings provide new insight into the modulation of rumen bacteriome caused by a mixed phytogenic feed additive in water buffaloes.

## Data Availability Statement

The datasets presented in this study can be found in online repositories. The names of the repository/repositories and accession number(s) can be found below: https://www.ncbi.nlm.nih.gov/, no. PRJNA564158.

## Ethics Statement

The animal study was reviewed and approved by Ethics committee of the Chinese Academy of Agriculture Sciences, Guangxi Buffalo Research Institute, China.

## Author Contributions

FH and CY: conceptualization. HE and ZT: data curation. FH, ZT, and HE: formal analysis. CY: funding acquisition, supervision, and validation. FH: investigation and writing–original draft. FH, HE, ML, LP, KP, and XL: methodology. XL and CY: project administration. ZT, ML, LP, KP, and CY: resources. FH and HE: software. FH, HE, and CY: writing–review and editing. All authors contributed to the article and approved the submitted version.

## Conflict of Interest

The authors declare that the research was conducted in the absence of any commercial or financial relationships that could be construed as a potential conflict of interest.
