# Supplementary material for: A Mixed Phytogenic Modulates the Rumen Bacteria Composition and Milk Fatty Acid Profile of Water Buffaloes
Source: Front Vet Sci. 2020 Aug 26;7:569. doi: 10.3389/fvets.2020.00569 (PMC7479126; doi:10.3389/fvets.2020.00569)
Supplement: Supplementary file 1 [file Data_Sheet_1.docx]

**Table S1. Phytochemical composition of mixture of phytonutrients**

| **Phytochemicals** | **Quantity** |
| --- | --- |
| Total Polyphenols (mg/g) as Gallic acid equivalent (GAE) | 12.65 |
| Total tannins (mg/g) as Tannic acid equivalent (TAE) | 9.62 |
| Total flavonoids (mg/g) as Catechin equivalent (CE) | 4.09 |

**Table S2. Relative abundance of bacterial phyla across different treatment groups**

| **Phylum name** | **Control** | **MP15** | **MP25** | **MP35** | **Corrected P-value** |
| --- | --- | --- | --- | --- | --- |
| Bacteroidetes | 65.07 | 61.16 | 58.45 | 51.57 | 0.17 |
| Firmicutes | 26.03 | 25.32 | 29.77 | 34.39 | 0.50 |
| Spirochaetes | 2.46 | 4.80 | 4.12 | 4.05 | 0.54 |
| Proteobacteria | 2.12 | 2.01 | 3.66 | 4.23 | 0.58 |
| Cyanobacteria | 1.51 | 3.07 | 0.93 | 2.19 | 0.11 |
| Kiritimatiellaeota | 0.86 | 0.73 | 0.64 | 0.97 | 0.59 |
| Fibrobacteres | 0.17 | 0.94 | 0.56 | 0.65 | 0.15 |
| Tenericutes | 0.41 | 0.47 | 0.65 | 0.65 | 0.08 |
| Patescibacteria | 0.37 | 0.78 | 0.40 | 0.59 | 0.21 |
| Synergistetes | 0.31 | 0.16 | 0.33 | 0.13 | 0.72 |
| Lentisphaerae | 0.27 | 0.16 | 0.21 | 0.20 | 0.35 |
| Actinobacteria | 0.12 | 0.15 | 0.15 | 0.16 | 0.88 |
| norank_d__Bacteria | 0.13 | 0.11 | 0.06 | 0.11 | 0.22 |

**Table S3. Relative abundance of bacterial genera across different treatment groups**

| **Species name** | **Control** | **MP15** | **MP25** | **MP35** | **Corrected**  **P-value** |
| --- | --- | --- | --- | --- | --- |
| Prevotella_1 | 49.24 | 44.98 | 39.96 | 31.76 | 0.66 |
| unclassified_o__Clostridiales | 4.96 | 5.74 | 5.53 | 3.62 | 0.84 |
| norank_f__F082 | 3.45 | 3.09 | 4.01 | 4.40 | 0.78 |
| Treponema_2 | 2.23 | 4.51 | 3.96 | 3.86 | 0.73 |
| Rikenellaceae_RC9_gut_group | 3.09 | 2.99 | 3.95 | 4.01 | 0.66 |
| Prevotellaceae_UCG-001 | 2.09 | 2.04 | 2.04 | 3.11 | 0.90 |
| Christensenellaceae_R-7_group | 2.07 | 1.80 | 2.39 | 2.33 | 0.85 |
| o__Gastranaerophilales | 1.51 | 3.07 | 0.93 | 2.19 | 0.66 |
| Succiniclasticum | 3.55 | 1.63 | 1.23 | 0.94 | 0.75 |
| Butyrivibrio_2 | 1.15 | 1.12 | 1.49 | 3.44 | 0.66 |
| Prevotellaceae_UCG-003 | 2.04 | 1.96 | 1.55 | 1.26 | 0.76 |
| f__Muribaculaceae | 1.03 | 1.13 | 2.19 | 2.13 | 0.90 |
| Ruminococcaceae_NK4A214_group | 1.49 | 1.11 | 1.99 | 1.58 | 0.66 |
| unclassified_f__Lachnospiraceae | 0.94 | 1.14 | 1.45 | 2.36 | 0.66 |
| Lachnospiraceae_XPB1014_group | 0.86 | 0.93 | 1.16 | 1.69 | 0.73 |
| Succinivibrionaceae_UCG-002 | 1.17 | 0.66 | 0.41 | 2.33 | 0.66 |
| Saccharofermentans | 0.92 | 0.76 | 1.03 | 1.67 | 0.66 |
| unclassified_f__Prevotellaceae | 0.64 | 1.48 | 1.15 | 1.07 | 0.66 |
| Pseudobutyrivibrio | 0.80 | 0.61 | 0.99 | 1.43 | 0.66 |
| o__WCHB1-41 | 0.86 | 0.73 | 0.64 | 0.97 | 0.76 |
| f__Bacteroidales_RF16_group | 0.51 | 0.44 | 0.89 | 1.16 | 0.75 |
| Ruminococcaceae_UCG-014 | 0.54 | 0.85 | 0.66 | 0.69 | 0.66 |
| Ruminococcus_1 | 0.37 | 0.56 | 0.72 | 1.06 | 0.66 |
| f__Bacteroidales_UCG-001 | 0.95 | 0.72 | 0.45 | 0.54 | 0.66 |
| Lachnospiraceae_NK4A136_group | 0.42 | 0.48 | 0.42 | 1.24 | 0.66 |
| Lachnospiraceae_AC2044_group | 0.48 | 0.52 | 0.65 | 0.88 | 0.66 |
| f__Lachnospiraceae | 0.36 | 0.47 | 0.59 | 0.98 | 0.66 |
| Fibrobacter | 0.17 | 0.94 | 0.56 | 0.65 | 0.66 |
| Eubacterium_coprostanoligenes_group | 0.40 | 0.57 | 0.80 | 0.34 | 0.66 |
| Ruminococcaceae_UCG-005 | 0.41 | 0.46 | 0.65 | 0.59 | 0.66 |
| Ruminococcaceae_UCG-010 | 0.41 | 0.49 | 0.62 | 0.55 | 0.83 |
| Eubacterium_ruminantium_group | 0.37 | 0.38 | 0.50 | 0.67 | 0.66 |
| Veillonellaceae_UCG-001 | 0.68 | 0.49 | 0.26 | 0.37 | 0.76 |
| Lachnospiraceae_ND3007_group | 0.26 | 0.31 | 0.47 | 0.65 | 0.66 |
| probable_genus_10 | 0.24 | 0.28 | 0.35 | 0.83 | 0.66 |
| f__Bacteroidales_BS11_gut_group | 0.31 | 0.31 | 0.42 | 0.60 | 0.66 |
| f__p-251-o5 | 0.36 | 0.44 | 0.38 | 0.37 | 0.90 |
| Acetobacter | 0.06 | 0.16 | 0.94 | 0.33 | 0.66 |
| Lachnospiraceae_NK3A20_group | 0.26 | 0.24 | 0.41 | 0.47 | 0.66 |
| Candidatus_Saccharimonas | 0.24 | 0.32 | 0.23 | 0.37 | 0.66 |
| Anaerovibrio | 0.36 | 0.26 | 0.15 | 0.32 | 0.73 |
| norank_o__Mollicutes_RF39 | 0.24 | 0.25 | 0.26 | 0.34 | 0.85 |
| Papillibacter | 0.16 | 0.25 | 0.32 | 0.31 | 0.66 |
| Ruminobacter | 0.17 | 0.25 | 0.21 | 0.40 | 0.85 |
| Prevotellaceae_YAB2003_group | 0.26 | 0.28 | 0.21 | 0.27 | 0.78 |

**Table S4. Correlation of bacterial genera with rumen fermentation parameters**

| **Bacterial Genus** | **Acetate** | **Propionate** | **isobutyrate** | **Butyrate** | **isovalerate** | **Valerate** | **A/P** | **TVFAs** | **NH_3_-N** |
| --- | --- | --- | --- | --- | --- | --- | --- | --- | --- |
| norank_f__F082 |  | -0.73 |  | -0.61 |  | -0.61 |  | -0.65 |  |
| Treponema_2 | -0.59 |  |  |  |  |  |  |  | 0.60 |
| Rikenellaceae_RC9_gut_group |  | -0.69 |  | -0.67 |  | -0.63 |  | -0.67 |  |
| Prevotellaceae_UCG-001 |  | -0.75 |  | -0.67 |  | -0.60 |  |  |  |
| unclassified_f__Prevotellaceae |  |  |  |  |  |  |  |  | 0.60 |
| Succiniclasticum |  |  | 0.60 |  |  |  |  |  |  |
| norank_f__Muribaculaceae |  |  |  |  |  |  | 0.71 |  |  |
| Ruminococcaceae_NK4A214_group |  | -0.38 |  |  |  |  |  |  |  |
| Lachnospiraceae_AC2044_group |  | -0.64 |  | -0.62 |  | -0.59 |  |  |  |
| Fibrobacter | -0.66 |  |  |  |  |  |  |  | 0.59 |
| Ruminococcaceae_UCG-005 |  | -0.64 |  | -0.70 | -0.59 | -0.63 |  | -0.66 |  |
| Lachnospiraceae_ND3007_group |  | -0.57 |  | -0.60 |  |  |  |  |  |
| probable_genus_10 |  | -0.66 |  | -0.58 |  |  |  |  |  |
| Candidatus_Saccharimonas | -0.64 | -0.73 |  |  |  |  |  | -0.69 |  |

Only significant (R>0.3 and P < 0.05) correlations are presented.

A/P=Acetate to propionate ratio; TVFAs=Total volatile fatty acids; NH_3_-N=Ammonia nitrogen

**Table S5. Correlation of bacterial genera with milk yield parameters**

| **Bacterial Genus** | **Milk Yield** | **Protein (%)** | **Protein Yield** | **Fat yield** | **Lactose** | **DMI** |
| --- | --- | --- | --- | --- | --- | --- |
| o__Clostridiales | 0.59 |  |  |  |  |  |
| Butyrivibrio_2 | 0.59 |  |  | 0.69 |  |  |
| Prevotellaceae_UCG-003 | -0.61 |  |  |  |  |  |
| Ruminococcaceae_NK4A214_group |  | -0.64 |  |  |  |  |
| Lachnospiraceae_XPB1014_group |  |  |  | 0.70 | -0.78 |  |
| Succinivibrionaceae_UCG-002 |  |  |  |  |  | 0.63 |
| Pseudobutyrivibrio | 0.67 |  | 0.61 | 0.68 |  |  |
| Ruminococcus_1 |  |  |  |  |  | 0.65 |
| Lachnospiraceae_NK4A136_group |  |  |  |  |  | 0.60 |
| Fibrobacter |  |  |  |  |  | 0.65 |
| Acetobacter |  |  |  |  |  | 0.62 |
| Lachnospiraceae_NK3A20_group | 0.58 |  |  | 0.64 |  |  |

Only significant correlations (R>0.3 and *P* < 0.05) are presented.
